# Antibacterial Activity of Ciprofloxacin-Encapsulated Cockle Shells Calcium Carbonate (Aragonite) Nanoparticles and Its Biocompatability in Macrophage J774A.1

**DOI:** 10.3390/ijms17050713

**Published:** 2016-05-19

**Authors:** Tijani Isa, Zuki Abu Bakar Zakaria, Yaya Rukayadi, Mohd Noor Mohd Hezmee, Alhaji Zubair Jaji, Mustapha Umar Imam, Nahidah Ibrahim Hammadi, Saffanah Khuder Mahmood

**Affiliations:** 1 Laboratory of Molecular Biomedicine, Institute of Bioscience, Universiti Putra Malaysia, Serdang, Selangor 43400, Malaysia; tjelyakub@gmail.com (T.I.); mustyimam@gmail.com (M.U.I.); 2 Faculty of Food Science and Technology and Laboratory of Natural Product, Institute of Bioscience, Universiti Putra Malaysia, Serdang, Selangor 43400, Malaysia; yaya_rukayadi@upm.edu.my; 3 Faculty of Veterinary Medicine, Universiti Putra Malaysia, Serdang, Selangor 43400, Malaysia; jajidvm@yahoo.com (A.Z.J.); naheda_ibrahem@yahoo.com (N.I.H.); saffanh.jeber@gmail.com (S.K.M.); 4 Laboratory of Pharmacology and Toxicology, Faculty of Veterinary Medicine, Universiti Putra Malaysia, Serdang, Selangor 43400, Malaysia; hezmee@upm.edu.my

**Keywords:** antimicrobial resistance, calcium carbonate (aragonite) nanoparticles, ciprofloxacin, intracellular infection, proinflammatory cytokine

## Abstract

The use of nanoparticle delivery systems to enhance intracellular penetration of antibiotics and their retention time is becoming popular. The challenge, however, is that the interaction of nanoparticles with biological systems at the cellular level must be established prior to biomedical applications. Ciprofloxacin–cockle shells-derived calcium carbonate (aragonite) nanoparticles (C-CSCCAN) were developed and characterized. Antibacterial activity was determined using a modified disc diffusion protocol on *Salmonella* Typhimurium (*S.* Typhimurium). Biocompatibilittes with macrophage were evaluated using the 3-(4,5-Dimethylthiazol-2-yl)-2,5-diphenyltetrazolium bromide (MTT) and 5-Bromo-2′-deoxyuridine (BrdU) assays. Transcriptional regulation of interleukin 1 beta (IL-1β) was determined using reverse transcriptase-polymerase chain reaction (RT-PCR). C-CSCCAN were spherical in shape, with particle sizes ranging from 11.93 to 22.12 nm. Encapsulation efficiency (EE) and loading content (LC) were 99.5% and 5.9%, respectively, with negative ζ potential. X-ray diffraction patterns revealed strong crystallizations and purity in the formulations. The mean diameter of inhibition zone was 18.6 ± 0.5 mm, which was better than ciprofloxacin alone (11.7 ± 0.9 mm). Study of biocompatability established the cytocompatability of the delivery system without upregulation of IL-1β. The results indicated that ciprofloxacin–nanoparticles enhanced the antibacterial efficacy of the antibiotic, and could act as a suitable delivery system against intracellular infections.

## 1. Introduction

Antimicrobial resistance is a growing problem [1]. The emergence of intracellular bacterial infections and acquired resistance of pathogenic microbes pose significant challenges for many antimicrobial agents [2]. Since the 1980s, flouroquinolones have been used in clinical practice [3], and they have contributed to major advances in the medical treatment of gram negative bacterial infections as frontline drugs [2]. Active efflux from prokaryotes as well as eukaryotic cells strongly modulates the activity of this class of antibiotics [4,5,6]. Thus, the intracellular actions of flouroquinolones are often sub-optimal [7] due to continued efflux [5,6]. This contributes to failure of conventional fluoroquinolones therapies as a result of decreased accumulation and poor retention of the antibiotics inside the cells [8,9]. Other factors limiting the success and clinical use of fluoroquinolones like ciprofloxacin include their bitter taste in solution, and rapid renal clearance, in which a minimum of 70% of the oral dose is excreted unchanged in the urine. Moreover, frequent administration of ciprofloxacin is associated with numerous side-effects [10,11,12]. In order to achieve successful treatment, antibiotics must fulfill a series of criteria, including the ability to penetrate and be retained by the cell, the capacity to reach the intracellular target, and the display of activity against bacteria residing in the intracellular environment [13]. On the other hand, due to the deficiency in new antibacterial agents, there is considerable interest in restoring the activity of older and conventional antimicrobials [14].

The use of safe and efficient delivery systems, capable of delivering therapeutic agents in an adequate concentration within the appropriate intracellular compartment is an ultimate goal in enhancing therapeutic effect. It is also a promising strategy in overcoming microbial resistance [15,16]. The encapsulation of antibiotics in carriers could avoid antibiotic efflux and enhance the drugs’ intracellular retention, since delivery systems like nanoparticles are not substrates of the efflux pump proteins [17]. Moreover, encapsulation of antibiotics improves their pharmacokinetics by increasing serum half-life [2]. Nanoparticles can be phagocytose by host phagocytes containing intracellular microbes. Once inside host phagocytes, the antibiotic–nanoparticle delivery system could release high dose of the antibiotic to eliminate the intracellular microbes before developing resistance [18,19,20].

Many studies have reported the increased antimicrobial activity of ciprofloxacin conjugated nanoparticles [21,22,23]. Likewise, decreased antibiotic resistance and increase antibacterial activity of ciprofloxacin was reported in the presence of Zinc Oxide nanoparticles [24]. It is anticipated that the use of nanoparticles-based drug delivery systems will continue to improve treatment of bacterial infections and multidrug-resistant microbes [19]. However, no studies have been conducted on the potential of ciprofloxacin encapsulated cockle shells-calcium carbonate (aragonite) nanoparticles (CSCCAN), to enhance the efficacy of the drug. The cockle shells (*Anadara granosa*), which is available in abundance, is often considered a waste and it is a cheap protein source [25]. Moreover, calcium carbonate has been used for controlled delivery of biomolecules due to it biodegradability, biocompatibility, porous nature and simple bulk-scale preparation [26,27]. A porous aragonite calcium carbonate nanoparticles loaded with gentamicin sulfate with controlled released property have been successfully used in osteomyelitis treatment [28]. Thus, calcium carbonate nanoparticles are expected to also enhance the efficacy of ciprofloxacin.

The continuous assembly of engineered nanoparticles as drug carrier system necessitates a comprehensive understanding of their potential toxicity [29]. Despite many reports on the toxicity of nanomaterials, the precise association between engineered nanoparticles and the immune system have not been broadly studied [29,30,31]. Macrophages are the key players in the innate immune response that phagocytose large foreign particles or endocytose biological molecules and tiny materials. Furthermore, engulfment of foreign materials renders macrophages more activated to complete the task of instigating and inducing the adaptive immune responses by discharging a variety of pro-inflammatory cytokine [32]. A critical indicator of nanoparticles toxicity is its ability to provoke an unwanted immune response in a biological system. Additionally, understanding the biological response to nanoparticles at the sub cellular level is crucial and can present further evidence on the interaction between nanomaterials and cells. It is thus essential to understand the immunogenic potential of the CSCCAN with respect to pro-inflammatory protein production.

## 2. Results and Discussion

### 2.1. Preparation of Nanoparticles

**Figure 1:**
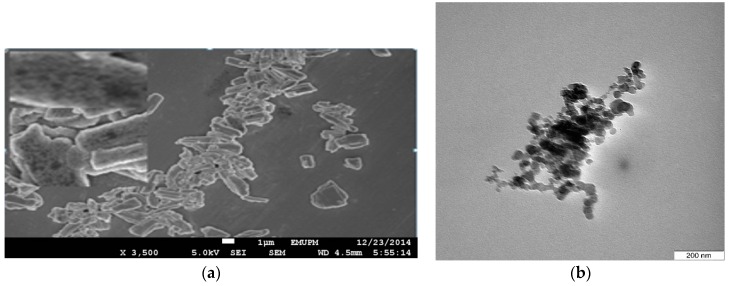
Field Emission Scanning Electron Microscope (FESEM) micrograph showing the pore structure of the micron-size cockle shells calcium carbonate powder, scale bar = 1 µm (**a**); and Transmission Electron Microscopy (TEM) micrographs showing nanoscale spherical-shaped cockle shells calcium carbonate (aragonite) nanoparticles (**b**).

The nanoparticles were synthesized in an optimized experiment via a microemulsion system using a high pressure homogenizer (HPH). Microemulsion system is recognized as the most ideal medium for the preparation of inorganic crystal particles [33]. In this study, the nanoparticles were successfully produced from a suspension of pore structured micron-sized cockle shells-calcium carbonate powder (CSCCP) (Figure 1a). As revealed by the Transmission Electron Microscopy (TEM) micrograph (Figure 1b), spherical-shaped nanoparticles were obtained during the synthesis. The average particles size was found in the range of 11.93 to 22.12 nm. 

During the synthesis of inorganic CaCO_3_, the presence of chemical modifiers, temperature, supersaturation, and pH are essential growth controlling factors [34]. It is likely that the amount of surfactant and co-surfactant (Tween-80 and glycerol) at the weight/volume ratio of 2:1 aided in the formation of the homogenous, nano-sized particles. Using Tween-80, Zou and coworkers (2009), synthesized homogenous nanoparticles with a smaller particle size and without aggregation, which was attributed to the superior emulsifying capacity of the surfactant [35].

Homogenizer is a proficient machine for the reduction of particle and droplet sizes [27]. The impact of high turbulence and shear force, coupled with compression and acceleration caused the breakdown of particles and dispersion throughout the sample [36]. After a 25-cycle homogenization process, the particles were evenly distributed in size, probably due to the number of cycles, and the operating pressure. Therefore, it is suggested that nanoparticles size and morphological structure can be controlled by adjusting the reaction medium and homogenization time.

### 2.2. Ciprofloxacin-Loading and Encapsulation Efficiency

The nanoparticles loading of drug is commonly accepted as the sum total amount of bounded drug per polymer mass [37]. The C-CSCCAN was successfully prepared in drug: nanoparticles ratio (1:17). The percentage LC of ciprofloxacin in the formulations was approximately 5.9% and the encapsulation efficiency (EE) was 99.5% The loading of ciprofloxacin into capsule material was suggested as a function of feeding concentration [38]. With the increase in the feeding concentration of ciprofloxacin, high loading capacity was observed. At a lower feeding concentration, the loading content became low. The small molecular size of ciprofloxacin [38], and the long overnight loading period was assumed to influence more loading. The increased loading capacity could also be induced by the porous nature of the nanoparticles (as shown in Figure 1a).

Encapsulation of various biomolecules into the hollow cavity of capsules could be achieved by several methods [39,40,41]. However, the present method is effortless and effective. The semi-water-soluble ciprofloxacin was probably deposited in the interior of the nanoparticles in a concentration dependent manner upon simple mixing and overnight stirring. The higher percentage encapsulation efficiencies were obtained with increasing amount of ciprofloxacin. This demonstrated minimal loss of the drug during the loading process. The above finding was similar to the reports presented previously [42,43]. Comparing the effectiveness of preparation methods, Abreu *et al.* (2010) observed that particles produced in water-in-oil emulsion method presented relatively higher EE values [44].

### 2.3. Characterization and Stability of Ciprofloxacin–Nanoparticles

**Figure 2:**
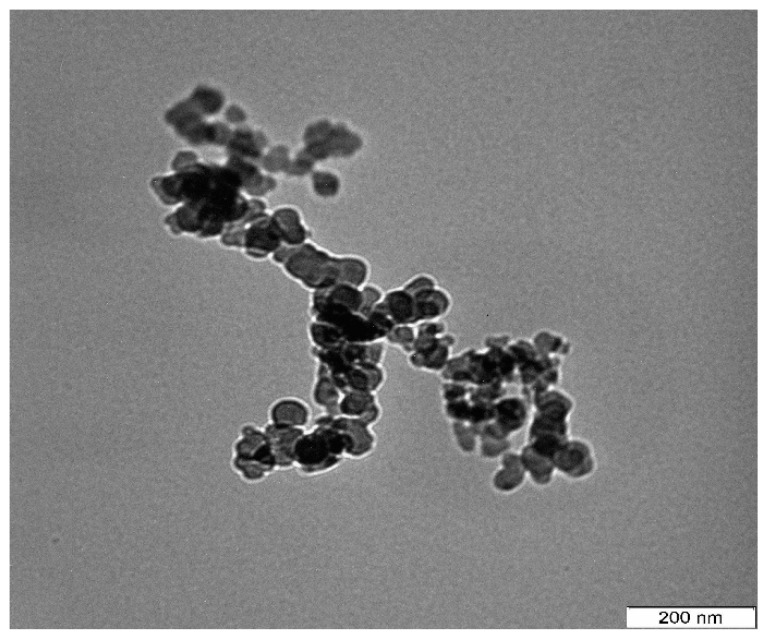
TEM micrograph of spherical shaped ciprofloxacin-encapsulated cockle shells calcium carbonate (aragonite) nanoparticles.

The comprehensive examination of physicochemical properties of nanoparticles is a prerequisite to full understanding of their potential application [45]. The micrograph images at lower magnification revealed the unaltered spherical shape of the nanoparticles (Figure 2) following the drug loading process. The particle sizes were between 13.94 and 23.95 nm and uniformly distributed. The particle sizes of nude nanoparticles (Figure 1b) were found to be the same as ciprofloxacin-encapsulated nanoparticles.

This result is in agreement with the observation of Mu and Feng (2003), who established that loading of drug into nanoparticles did not significantly affect the particle size [46]. However, it has been visibly demonstrated that size of nanoparticle is one of the most essential feature in influencing the activities of the complexes *in vitro* and *in vivo* [47], and it plays a significant role in the uptake by macrophages and passive penetration of the cell membrane [48,49]. Therefore, the reduced particle size obtained in this study was suggested to improve patient comfort.

**Figure 3:**
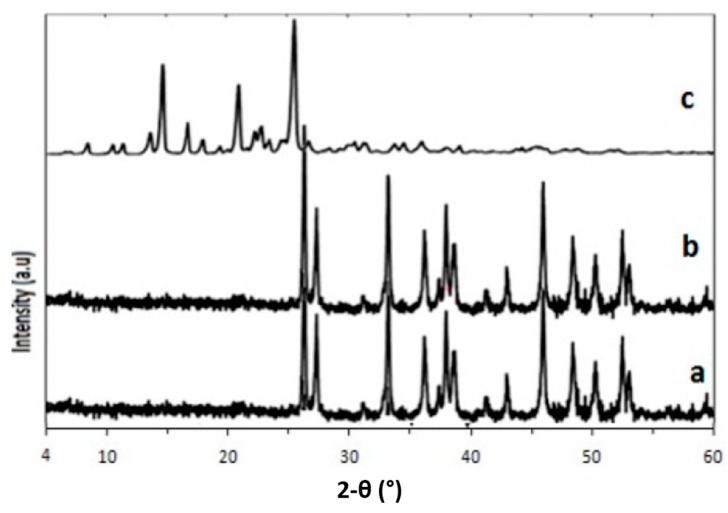
X-ray Powder Diffraction (XRD) spectra of cockle shells calcium carbonate (aragonite) nanoparticles (**a**); ciprofloxacin-encapsulated cockle shells calcium carbonate (aragonite) nanoparticles (**b**); and free ciprofloxacin (**c**) showing crystalline phases and purity.

The characteristic crystalline structure and purity of the samples were estimated using X-ray diffraction (Figure 3a–c). Strong crystallizations were observed in the formulation, as indicated in Figure 3a,b, corresponding to CSCCAN and C-CSCCAN, respectively. Broad sharp, crystal peaks were observed at diffraction angles of 2θ = 26.2, 27.3, 33.1, 38.7, 38.8, 46.1, 48.3, and 52.8 in all the samples.

The original crystal natures of CSCCP were not changed and did not disappear during preparation and when loaded with ciprofloxacin. Likewise, several sharp and clear X-ray Powder Diffraction (XRD) diffraction peaks attributable to ciprofloxacin could not be traced in the loaded nanoparticles, suggesting that the preparation process has no effect on the nanoparticles crystalline properties and the formulation is free from impurities. This observation was supported by an earlier finding [50]. Ciprofloxacin showed its specific crystal peaks around 2θ = 14.1, 21.1, and 25.1. Likewise, several sharp and clear XRD diffraction peaks attributable to ciprofloxacin could not be traced in the loaded nanoparticles.

The ζ potential is usually an indicator of the stability of colloidal dispersion, which measures shelf life, nanoparticle interaction with charged drugs in a dispersion, and also the bonding and electrostatic repulsion of drug delivery systems with biological system [51]. The ζ potentials of the formulations in pH 7.4 exhibited a slightly negative charge of −15.3 ± 2.0 mV and −13.0 ± 1.9 mV for the CSCCAN and C-CSCCAN, respectively. Evaluating the ζ potential values between the loaded nanoparticles and nude nanoparticles revealed that loading of the antibiotic slightly reduced the surface charge of the carrier. The differences in ζ potential may be due to physico-chemical properties of the drug. However, the strengths of the cationic drug like ciprofloxacin could be expected to influence formulations ζ potential only when the drug was present at the surface [52]. Since such an effect was not observed, nearly the sum total of the drug molecules was possibly incorporated into the inner core of the nanoparticles. The negative potential values indicate stability and electrostatic repulsion that protects the particles from aggregation to some extent, though Kanaujia and coworkers (2011) have stressed that higher negative or positive ζ potential prevents aggregation of the particles, due to electric repulsion and this electrically stabilizes the nanoparticles dispersion [53]. In both negatively and positively charged particles, experts have established that the degree of phagocytosis enhances with increasing ζ potential, and is low when ζ potential is zero [49]. Lu *et al.* (2007) stated that the half-life of encapsulated carrier might be increased in circulation when the carrier is negatively charged, since the surface charge of the endothelium is also negative [47]. Therefore, the optimal potential values obtained in the present study suggest the suitability of the nanoparticles for systemic drug delivery.

### 2.4. Biocompatibility Evaluation

#### 2.4.1. Immunogenicity Assessment

Immunogenicity is the capability of a certain substance to provoke immune response in biological systems. Unwanted immune response to therapeutic agents may result in adverse events such as systemic inflammatory response [54,55]. Immunogenicity is influenced by multiple characteristics of an antigen including molecular size, chemical composition and degradability. Analyzing the interaction of nanoparticles with the body defense mechanisms is of particular relevance in the event of the particles employed for medical applications, as they are often injected into the blood stream and can be found in direct contact with a large number of immune cells [56].

**Figure 4:**
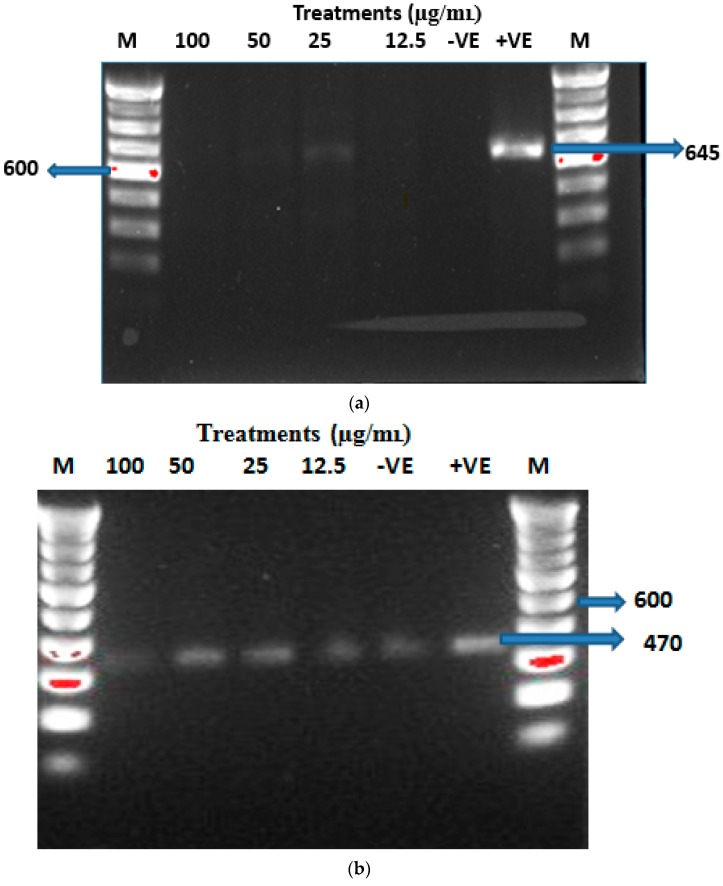
RT-PCR data showing IL-1β (**a**) and β-actin (loading control) (**b**), mRNA expressions after 3 h of CSCCAN treatment. −VE, negative control or untreated cells; +VE, positive control (Treated with Bacterial lipopolysaccharides); M, molecular weight markers (100 bp); DNA ladder MW, 600; IL-1β product size, 645; β-actin product size, 470.

In this study, the immunological response of macrophage cells subjected to CSCCAN was studied with regard to expression of pro-inflammatory cytokine, interleukin 1 beta (IL)-1β, after 3 h incubation (Figure 4a). The RT-PCR analysis revealed that cells treated with nanoparticle concentrations up to 100 µg/mL did not stimulate a significant increase in expression of IL1-β gene, which is associated with inflammation and toxicity signaling pathways *in vitro*. IL-1β mRNA was only detected in the cells treated with bacterial lipopolysaccharides (positive control). However, the intensity of the band and gene expression pattern for β-actin mRNA (loading control) (Figure 4b) was similar to that of IL-1β gene extracted from cells treated with bacterial lipopolysaccharides. This result implies that CSCCAN does not elicit an immediate immunological response nor does it stimulate the production of pro-inflammatory proteins, a critical indicator of toxicity [57].

#### 2.4.2. Viability Test

**Figure 5:**
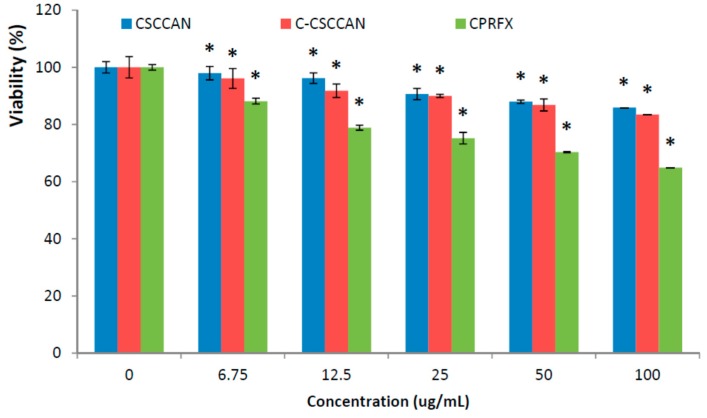
The MTT percentage viability of proliferating cells. The values represent mean ± standard deviation (=3); * (*p* < 0.05) compared with ciprofloxacin (CPRFX).

Addressing the cytotoxicity of the nano-antimicrobial agent against eukaryotic cell line prior to its intended application is an important step [58,59]. Macrophage cells treated with different concentrations of the nanoparticle formulations and ciprofloxacin (control), 100, 50, 25, 12.5 and 6.75 µg/mL, were assessed using MTT assay for cell-viability determination (Figure 5). After 24 h treatment, the macrophage J774.1A cells showed more than 80% viability at 100 µg/mL of CSCCAN and C-CSCCAN, using doses above minimum inhibitory concentrations for the bacteria tested in this study, which was better than that of free ciprofloxacin (*p* < 0.05). However, the cytotoxicity assay showed an insignificant concentration-dependent viability. The cell viability dropped slightly at the formulations concentration of 50 and 100 µg/mL. This observation indicates biocompatibility of CSCCAN and C-CSCCAN.

The comparison of the effect of the complex formulations on cell viability against free ciprofloxacin allows us to truly have a better insight into the complicated problem of toxicity. When free ciprofloxacin was used in similar doses, there was a considerable decrease in cell viability. This slight reduction in the cell number could be due to stress caused to the cells, signifying possible cytotoxic effect of the drug. Moreover, ciprofloxacin cytotoxic effects have been reported including its proapoptotic effects in Jurkat T cell *in vitro* [60], activation of monocytes and macrophages [61], and *in vitro* heritable genotoxic effects on human liver cells [62]. Ciprofloxacin was also found to stimulate cytotoxicity in human fibroblast cell cultures linked to oxidative stress [63]. The mechanism of experimental ciprofloxacin cytotoxicity has been correlated to selective reduction of mitochondrial-DNA in the course of an interference with a mitochondrial topoisomerase II-like activity [63,64].

The results in this study suggest biocompatibility of C-CSCCAN as a potential nano-antimicrobial agent and thus foretell its suitability for biological applications particularly in the systemic intracellular drug delivery. In addition, the choice of the 24 h incubation period was to mimic tissue-therapeutic contact time, which will be expected in an *in vivo* experiment where clearance (uptake) would take longer. Furthermore, the 24 h exposure was chosen since the cells could be within a logarithmic growth phase. In this period, any toxicity as a result to inhibition of proliferation and/or cell death, will be visibly in the MTT assay [65].

#### 2.4.3. Genotoxicity Test

Damage to DNA may occur through indirect mechanisms whereby the nanomaterials do not physically interact with the DNA molecule. Additionally, nanomaterials may induce other cellular responses that consequently result in genotoxicity, such as oxidative stress, inflammation and abnormal signaling responses [66]. 5-bromo-2′-deoxyuridine (BrdU) is a substitute of thymidine nucleotide that can be incorporated instead of the thymidine during DNA replication. Once DNA is damaged as a result of external stimuli, the BrdU is not integrated to the newly synthesized DNA during replication process signifying genotoxic effect [67].

**Figure 6:**
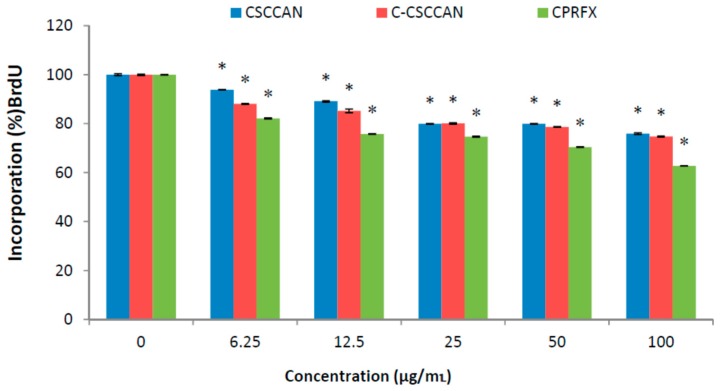
The percentage of BrdU incorporation into the DNA of proliferating cells. The values represent mean ± standard deviation (*n* = 3); * (*p* < 0.05) compared with ciprofloxacin.

The BrdU assay was used in the present study with the macrophages J774A.1 cells to evaluate DNA damage and genotoxicity to individual cell population. Cells were treated with different concentrations of the nanoparticles formulations and ciprofloxacin (positive control). The percentage of labeled precursor (BrdU) incorporation into the cells indicated that nanoparticles formulations caused insignificant DNA damage; hence, they were non-genotoxic (Figure 6). It also showed no considerable difference between the formulations at various treatment groups.

### 2.5. Antibacterial Activity

**Figure 7:**
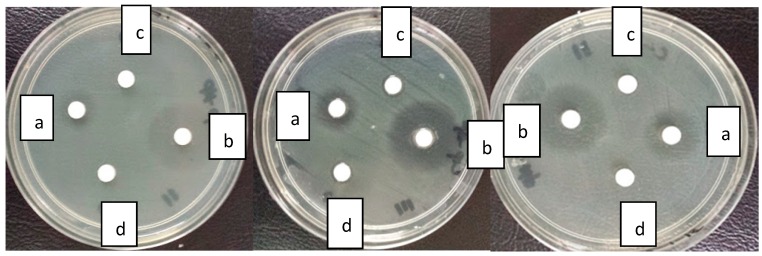
Disk diffusion assay displaying zone of inhibition diameter of ciprofloxacin (**a**); ciprofloxacin–cockle shells calcium carbonate aragonite nanoparticles (**b**); cockle shells calcium carbonate aragonite nanoparticles (**c**); and dimethylsulfoxide (**d**).

The diameter of inhibition zone reflects magnitude of susceptibility of microorganisms [68]. The antibacterial activities of C-CSCCAN and free ciprofloxacin against *S.* Typhimurium strain was evaluated using the impregnated disk diffusion method. The antibacterial activities of ciprofloxacin were increased in the presence of CSCCAN against the test strain. The mean diameter zones of inhibition (Table 1, Figure 7) were 18.6 ± 0.5 and 11.7 ± 0.9 for C-CSCCAN and ciprofloxacin impregnated disks, respectively. The former represent almost 48% greater efficiency than that observed with the latter. Conversely, the nude CSCCAN did not present any inhibition zone diameter value. In the case of the C-CSCCAN, as compared to ciprofloxacin, the mean diameter zone of inhibition values was increased significantly (*p* < 0.05).

The results suggest that while CSCCAN has no antibacterial activity against *S.* Typhimurium, its preparation with ciprofloxacin enhanced the antibacterial activity of the antibiotic. In a similar development, Banoee *et al.* (2009) reported an *in vitro* enhanced antibacterial activity of ciprofloxacin in combination with ZnO nanoparticles against *Staphylococcus aureus* and *Escherichia coli*, which was suggested to the direct nanoparticle interference with NorA protein pumping activity of the tested organisms [24]. Similarly, time dependent studies have demonstrated similar improvements in antimicrobial activity of ciprofloxacin encapsulated in carrier system, shortened the time of sterilization [38,69].

Many studies have described the enormous benefits with nano-particulate delivery systems, including the sustained release of antibiotic for prolonged durations, which in turn used to attain the minimum inhibitory concentration for extended time [51]. The data obtained in the present study may declare that encapsulated ciprofloxacin were successfully prepared and was continuously released from nanoparticles for a longer period, thus significantly extended the drug effect compare to the free ciprofloxacin. The higher the antibacterial effect of ciprofloxacin–nanoparticles may have resulted from higher diffusion of the nanoparticles through the bacteria. This indicates that the effective dose of this antibiotic can be reduced against *S.* Typhimurium, thereby reducing the side effects of the drug. It has been revealed that nanoparticles are able to be endocytose by phagocytic cells and release the drug into the cells [9,70]. The ciprofloxacin loaded nanoparticles could be useful in the targeting of the drug to the phagocytic cells to improve the treatment of intracellular infections compared to the treatment using free ciprofloxacin.

## 3. Materials and Methods

### 3.1. Reagents, Chemicals and Media

Polysorbate-Tween 80 (Thermo Fisher Scientific, Waltham, MA, USA), glyceride (Sigma-Aldrich Co., St. Louis, MO, USA), ciprofloxacin (LKT Laboratories, St. Paul, MN, USA), dulbecco’s modified eagle’s medium (Sigma-Aldrich Co.), foetal bovine serum (Sigma-Aldrich Co.), mueller–hinton agar (Difco Becton Dickinson, Sparks, MD, USA), mueller–hinton broth (Difco Becton Dickinson), ethanol (Joseph Mills Denaturants, Liverpool, UK), phosphate buffer saline (Sigma-Aldrich Co.), dimethylsulfoxide (Sigma-Aldrich Co.), MTT (3-(4,5-dimethylthiazol-2-yl)-2,5 diphenyltetrazolium bromide) dye (Naclai tesque, Inc., Kyoto, Japan), penicillin and streptomycin antibiotics (i-DNA Biotecnology(M) Sdn Bhd, Kuala Lumpur, Malaysia), Access RT-PCR System (Promega Corp., Madison, WI, USA), lipopolysaccharides from *Salmonella* enterica serotype enteritidis (Sigma-Aldrich Co.), RNA isolation kit (RBC Bioscience Corp., Xiandin Dist., New Taipei, Taiwan), BrdU cell proliferation assay kit (Biovision Inc., Milpitas, CA, USA), agarose LE, analytical grade (Promega Corp.), SYBR Safe DNA gel stain (Invitrogen, Waltham, MA, USA), loading dye and DNA ladder (i-DNA Biotechnology (M) Sdn Bhd).

### 3.2. Bacterial Strain

*Salmonella* Typhimurium ATCC 14208 was obtained from American Type Culture Collection (ATCC, Rockville, MD, USA). *S*. Typhimurium ATCC 14028 were grown and maintained on Tryptic Soy agar (TSA) (Sigma-Aldrich Co.).

### 3.3. Top-Down Synthesis of Cockle Shells Calcium Carbonate Nanoparticles

The synthesis of the nanoparticles was achieved via oil-in-water (O/W) microemulsions using a high pressure homogenizer (HPH) [71,72]. The microemulsion samples were prepared by mixing the surfactant and co-surfactant (Tween-80 and glycerol) into 20 mL oil in a glass reactor followed by addition of the 65 mL deionized water. The mixed solutions were stirred for 30 min until they became transparent. The nanoparticles were first prepared by suspending 2 gram of dry cockle shells-calcium carbonate powder (CSCCP) into the formulated oil-in-water (O/W) microemulsion, and moderately stirred for 10 min at 1000 rpm to form a cockle shells calcium carbonate suspension. The formulated suspension was sucked through the fluid opening tube of HPH for pre-milling at low pressures of 200 and 500 bars for three cycles each. After pre-milling, the suspension was collected at the HPH outlet and was again passed through the HPH fluid inlet at a high pressure of 1500 bars for 25 homogenizing cycles to get the desired fine particles. The final particles suspension was filtered and oven dried at 95 °C for 24 h [26].

### 3.4. Drug Loading and Encapsulation

An optimized aqueous solution of ciprofloxacin (3 mg/mL) was added into the CSCCAN suspension (50 mg/mL). The formulation was mechanically agitated overnight at 200 rpm at room temperature using a laboratory multi-hotplate stirrer (Witeg, Wise Stir SMHS, Witeg Labortechnik GmbH, Wertheim, Germany). Then it was centrifuged at 15,000 rpm for 15 min. The ciprofloxacin–cockle shell-derived calcium carbonate (aragonite) nanoparticles (C-CSCCAN) were freeze-dried [50].

The ciprofloxacin loading and encapsulation efficiency were analyzed by calculating the difference between the total drug (*Wt*) and the free encapsulated drug (*Wf*) in the nanoparticles supernatant per nanoparticles weight. The residual quantity of free encapsulated ciprofloxacin remaining in the supernatant was determined by computing the optical density at 291 nm [73], on a micro-titer plate reader (TECAN Safire, Tecan Austria GmbH, Grödig, Austria) [74]. A calibration curve of standard ciprofloxacin solution was used to obtain ciprofloxacin concentration (*R*^2^ = 0.9823, *Y* = 0.0091*X* + 1.4894). Data were given as an average measurement of three independent values. Drug loading contents (LC%) and encapsulation efficiency (EE%) of the nanoparticle were calculated as previously described [75]:

$$\text{Loading Content}=\frac{Wt-Wf}{\text{Wnp}}\times 100$$

where *Wt* = the total weight of drug used, *Wf* = the weight of non-encapsulated free drug, and Wnp = the weight of the nanoparticle.

$$\text{Encapsulation Efficiency}=\frac{Wt-Wf}{Wt}\times 100$$

where *Wt* = the total weight of drug, and *Wf* = the weight of non-encapsulated free drug.

### 3.5. Transmission Electron Microscopy

The shape and particle size of CSCCAN and C-CSCCAN were analyzed using Transmission Electron Microscopy (TEM) (Hitachi H-7100, Tokyo, Japan). Samples were mixed with 95% alcohol then sonicated for 45 min. The colloidal drop from each sample were loaded on carbon coated copper grids, placed on a filter paper, and dried at room temperature for 1 h. TEM measurements were carried out at 150 kilovolts [76].

### 3.6. Field Emission Scanning Electron Microscope

The surface morphology of nude CSCCAN and C-CSCCAN were observed with Field Emission Scanning Electron Microscope (FESEM) (Model 100, Perkin Elmer, 710 Bridge port Avenue, Shelton, CT, USA), equipped with an energy-dispersive X-ray spectroscopy. The samples were individually prepared on aluminum stubs and coated with gold under argon atmosphere using a sputter coater. The FESEM observations were performed at 200 kilovolts.

### 3.7. ζ Potential

The particle surface charge was measured using Malvern Zetasizer Nano (Malvern Instruments, Worcestershire, UK), which measures the electrophoretic mobility of particles in an electrical field, which is then converted into ζ potential. The measurement was performed by injecting the samples into the cells of the zetasizer and run at room temperature. The process was done three times and the average was taken to determine the ζ potentials.

### 3.8. X-ray Powder Diffraction

The purity and crystalline properties of the CSCCAN, C-CSCCAN and free ciprofloxacin powders were investigated using X-ray Powder Diffraction (XRD) (Shimadzu XRD-6000, Yokohama, Kanagawa, Japan). Cross-section of the samples was taken and placed on a quartz plate for exposure to Cu Kα radiation of wavelength λ = 1.5406 Å. The samples were then examined at room temperature over a 2θ range of 4°–50°, with sampling intervals of 0.02° 2θ and a scanning rate of 0.6°/min.

### 3.9. In Vitro Biocompatibility Assays

#### 3.9.1. Cell Culture

Macrophage J774A.1 cells were purchased from the American Type Culture Collection (ATCC). They were maintained as semi-adherent cell cultures at 37 °C in a humidified atmosphere (5% CO_2_) in Dulbecco modified Eagle’s minimal essential medium (DMEM, 25 mM glucose) supplemented with 10% heat-inactivated foetal bovine serum (FBS) and 100 µg/mL each of penicillin and streptomycin.

#### 3.9.2. RNA Extraction and RT-PCR

Macrophages J774A.1 cells were seeded at a density of 5 × 10^5^ cells/well in 2 mL medium of 6-wells culture plates and grown overnight. The cells were then washed with PBS and treated with different concentrations of CSCCAN in culture medium suspension (100–3.125 µg/mL) for 3 h. Bacterial lipopolysacchride treated cells served as a positive control, and untreated cells served as a standard control for RT-PCR analysis. After exposure to nanoparticles, cells were collected, extracted and analyzed for mRNA expression of IL-1β cytokine. The pro-inflammatory cytokine, IL-1β, was evaluated and β-actin was used as the control. The total RNA was extracted using the RBC Bioscience Total RNA Isolation kit, according to the manufacturer’s instructions. RNA Quantification and purity was determined using NanoDrop Spectrophotometer. Primer sequences were designed on the NCBI website with accession number: NM_008361.3 and product size: 645 for IL-1β and β-actin with accession number: NM_013458.5 and product size: 470. The primers (Table 2) were supplied by (First Base Laboratories Sdn Bhd, Kuala Lumpur, Malaysia). Reverse transcription-PCR were performed according to the Access Reverse Transcriptase System Protocol in a sensquest labcycler under the following conditions: 45 °C for 45 min, 94 °C for 2 min, 94 °C for 30 s, 65 °C for 1 min, 72 °C for 2 min, and 68 °C for 7 min for a total of 40 cycles. The same cycling conditions were used for β-actin with the exception of annealing temperature (59 °C for 1 min). The PCR amplification products were analyzed by gel electrophoresis on a 0.1% agarose gel, stained with SYBR Safe DNA Gel Stain, and fluoresce with ultraviolet source.

#### 3.9.3. MTT—Viability Assay

For cytocompatibility study, macrophages J774A.1 were split by mechanical scraping and cell suspensions were seeded in 96-well micro-titer plate at a density of 10,000 cells/well and incubated for 24 h. During each experiment, the media was removed and the cells were cultured with 100 µL of different concentrations of C-CSCCAN, CSCCAN and ciprofloxacin in culture medium (100 to 6.25 µg/mL), or of the control (culture medium only). The experiment was conducted in triplicates, and the optical densities were measured at 570 nm in a micro-titer plate reader [50,74]. The cell viability was computed using the following equation:

Cell Viability (%) = A_test_/A_control_ × 100

where A_test_ is the optical density of the cells incubated with the different treatments and A_control_ is the optical density of the cells incubated with the culture medium only (negative control). The cytotoxicity was determined from the average of three replicate tests and results were expressed as mean ± standard deviation.

#### 3.9.4. BrdU (ELISA) Genotoxicity Assay

The cell genotoxicity and quantitative determination of DNA synthesis in cells was evaluated based on the incorporation of BrdU into the synthesized DNA of a proliferating cell [67]. Macrophages J774A.1 cells were seeded into 96-well micro-titer plate at density at a density of 1 × 10^4^ cells per well and incubated for 24 h. The cells were then washed with PBS and treated with different concentrations of C-CSCCAN, CSCCAN and ciprofloxacin in culture medium suspension (100–3.125 µg/mL). Cells incubated with culture medium only (untreated cells) were regarded as negative control. After the cell incubation for 24 h, the medium was removed and the BrdU labeling assay was performed according to the manufacturer’s instructions (Bio Vision Incorporated, Milpitas, CA, USA). The color intensity absorbance directly correlated to the amount of synthesized DNA, which also reflects the number of proliferating cells. The BrdU incorporation was determined by analyzing cells treated compared to controls and the absorbance was measured at 450 nm using a micro-titer plate reader [67]. The data were determined from the average of three replicate tests and results were expressed as mean ± standard deviation.

### 3.10. In Vitro Antibacterial Susceptibility Test

The bacterial stock solution were diluted according to the method in Clinical and Laboratory Standards Institute (CLSI) [77].

#### 3.10.1. Preparation of Drugs Stock Solutions

A stock solution of C-CSCCAN suspensions and ciprofloxacin dispersion (pH 7.4) at concentration of 1 mg/mL was prepared in 10% DMSO.

#### 3.10.2. Disc Diffusion Susceptibility Assay

Susceptibility of C-CSCCAN and ciprofloxacin against *S.* Typhimurium were determined by disc diffusion method described previously [78], with little modifications. This method was performed in Muller Hinton agar media. A single colony of *S.* Typhimurium was grown overnight in MHB on a rotary shaker (250 rpm) at 35 °C. Inoculums were prepared by diluting the overnight cultures to a 10^6^ colony-forming units/mL (CFU/mL) suspension according to the turbidity of 0.5 McFarland standards. Then, 100 µL bacterial suspensions were inoculated to the prepared MHA plates and spread all over in one direction. Sterile paper disc (6 mm in diameter) was placed on the MHA plates and impregnated with 10 µL of C-CSCCAN formulation and CSCCAN (100 µg/mL). Ciprofloxacin (100 µg/mL) and DMSO was correspondingly prepared and served as a positive and negative control respectively. After 37 °C incubation for 24 h, the diameters of inhibition zone were measured in millimeters. The data obtained from three replicate tests were expressed as mean ± SD.

### 3.11. Statistical Analysis

All statistical analyses were performed using Minitab statistical software (Minitab Inc., State College, PA, USA). All experiments were performed in triplicate. Values were expressed as mean ± standard deviation. Comparisons of treatment effects and statistical significant differences between groups were determined using one-way analysis of variance (ANOVA) (for MTT and BrdU Assay) and Student’s independence *t*-test (for disc diffusion assay). A value of *p* < 0.05 was regarded significant unless indicated otherwise.

## 4. Conclusions

The aim of nano-antibacterial therapy is to achieve delivery of drug molecules, especially in the sub-cellular organelles. This study shows that the physicochemical properties of CSCCAN were the major factors influencing the antibacterial performance and increased susceptibility of *S.* Typhimurium. The encapsulation and subsequent sustained slow released of ciprofloxacin possibly modified and increased the drug cellular permeation, half-life and reduced its efflux to allow maximum tolerated dose. The outcome of antibacterial study bears significant implications, because a reduction in dose frequency due to delivery of drug molecules inside the cells would indeed enhance patient compliance and the safety of treatment and it could offer a great possibility to overcome the emerging problem of antibiotic resistance among a range of disease causing bacteria. The present study has demonstrated the biocompatibilities (nontoxicity and nonimmunogenicity) of CSCCAN and its ciprofloxacin formulation, making them suitable candidates for nanomedicine and related fields.

## Figures and Tables

**Table 1:** Mean zone of inhibition (mm) of free ciprofloxacin, C-CSCCAN and CSCCAN suspension (10 µL).

Formulations	C-CSCCAN	Ciprofloxacin	CSCCAN
Tested Bact.	-	-	-
*S*. Typhimurium	18.6 ± 0.5	11.7 ± 0.9	N/I

The values represent mean ± standard deviation (*n* = 3); *p* > 0.05 compared with ciprofloxacin. N/I, No inhibition; C-CSCCAN, ciprofloxacin–encapsulated cockle shells calcium carbonate aragonite nanoparticles; CSCCAN, cockle shells calcium carbonate aragonite nanoparticles.

**Table 2:** Gene names and sequences of primers.

**Gene Name**	**IL-1β**
Forward Primer Sequences	GCTGCTTCCAAACCTTTGAC
Reverse Primer Sequence	GCTTGTGCTCTGCTTGTGAG
**Gene Name**	**β-Actin**
Forward Primer Sequences	CATGAGGCTTATATCCTTGC
Reverse Primer Sequence	TAAAAGGCACTTTGTCCACT
